# Comparative Process Mining for Identifying the Critical Activities in Sepsis Trajectories

**DOI:** 10.1049/htl2.70010

**Published:** 2025-05-05

**Authors:** Mohsen Mohammadi

**Affiliations:** ^1^ Computer Engineering Department Esfarayen University of Technology Esfarayen Iran

**Keywords:** critical activity, healthcare, process mining, sepsis trajectories

## Abstract

Sepsis, a life‐threatening condition with high mortality and readmission rates, demands precise and timely management to improve patient outcomes. Despite advancements, identifying the critical activities within sepsis treatment pathways remains a challenge, limiting the effectiveness of interventions. This study addresses this issue by utilising comparative process mining techniques to analyse sepsis trajectories, focusing on key performance metrics—sojourn time, arrival rate and finish rate—across distinct patient clusters. The analysis is based on real‐life event logs from a hospital's sepsis cases, using K‐means clustering to segment patients by age, severity and key clinical indicators. The study reveals critical activities such as ‘Return ER’, ‘Admission IC’, and ‘Release C’, which consistently exhibit high sojourn times and influence patient outcomes significantly. These activities emerge as bottlenecks in the patient care process, particularly in cases of severe sepsis, where delays can lead to increased complications and mortality. By identifying these critical points, the study provides actionable insights for healthcare providers to optimize resource allocation, reduce delays and enhance the overall efficiency of sepsis management. The findings underscore the importance of targeted interventions in these key areas, offering a path toward improved clinical outcomes and reduced sepsis‐related mortality and readmission rates. This research contributes to the growing field of process mining in healthcare, highlighting its potential to transform complex clinical pathways into more efficient and effective treatment processes.

## Graphical abstract

This paper employs comparative process mining techniques to analyze real‐life hospital event logs from sepsis cases, identifying critical activities that significantly influence patient outcomes. By pinpointing these key steps in sepsis treatment trajectories, the study provides actionable insights for optimizing clinical processes and improving healthcare delivery.

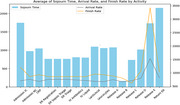

## 1 Introduction

The average mortality rate for sepsis in hospitals is approximately 35%. Out of every 1000 patients, around 10 are diagnosed with sepsis, with 30% of these cases leading to Multiple Organ Dysfunction Syndromes [1, 2]. Additionally, sepsis has a notably high readmission rate, with 18−26% of patients returning to the hospital within 30 days of discharge [2, 3]. The Systemic Inflammatory Response Syndrome (SIRS) criteria, including abnormal respiratory rate, heart rate, temperature and leukocyte count, are fundamental in identifying systemic inflammation, an early indicator of sepsis that can escalate to severe organ dysfunction and septic shock if not promptly addressed [4]. Organ dysfunction and hypotension are markers of severe sepsis, significantly increasing the risk of mortality [5]. Elevated lactic acid levels indicate tissue hypoperfusion, a critical marker for poor outcomes, while oliguria reflects kidney dysfunction, emphasising the importance of these factors in assessing and managing sepsis [6].

Timely diagnosis and appropriate patient care are crucial for effectively managing sepsis [7]. In this regard, innovations play a significant role in enhancing healthcare by making it more affordable and efficient. Advancements such as new technologies and business models are driving progress in the healthcare sector (Herzlinger, [8]). Moreover, healthcare systems globally are facing unprecedented challenges, including the need to rapidly adapt clinical processes based on emerging scientific evidence (Peiffer‐Smadja et al., [9]) and to deliver high‐quality care despite limited resources (Mans et al., [10]). Consequently, hospitals and other healthcare organisations recognise the importance of managing and improving their clinical processes [11]. In this context, process mining has emerged as an increasingly valuable method for understanding and optimising clinical processes by extracting insights from event logs generated by healthcare information systems. This approach merges data science with process management techniques, enabling healthcare organisations to visualise, analyse and enhance their processes (Munoz‐Gama et al., [12]).

Process mining techniques are employed to analyse business processes based on the data recorded during their execution. These methods are applied across various domains, including healthcare, where the focus is primarily on analysing diagnostic, treatment and organisational processes. Process variant analysis, which identifies differences between groups of process executions, aids in determining whether process improvements are necessary and, if so, what changes can enhance efficiency [11, 13]. Although prior research has demonstrated the effectiveness of process mining in analysing sepsis trajectories, the comparative analysis of these methods to pinpoint the most critical activities within sepsis treatment pathways remains insufficiently addressed. Therefore, the objective of this paper is to conduct a comparative analysis using process mining techniques to identify the most critical activities within sepsis treatment pathways. By leveraging data from sepsis event logs, the study aims to uncover variations and key factors that influence patient outcomes.

The remainder of this paper is structured as follows: Section 2 provides an overview of the background of process mining in the healthcare domain and reviews related works. Section 3 details the technique and its application to sepsis data. Section 4 discusses the findings from the case study. Finally, Section 5 presents a concise summary and highlights the ongoing potential and future directions of comparative process mining in healthcare.

## 2 Background and Related Works

### 2.1 Process Mining in Healthcare

Process mining techniques can be utilised to analyse business processes by examining the data recorded during their execution. These methods are applied across various fields, including healthcare, where they primarily focus on analysing diagnostic, treatment and organisational processes [11].

The data produced during the execution of healthcare processes are crucial assets for managing and enhancing these processes. Healthcare institutions frequently depend on Health Information Systems to record various data points during the execution of these processes. For example, such systems document when a patient is registered or undergoes a clinical examination by a physician. The information stored within HIS databases can be utilised to create event logs that chronicle the sequence of actions performed, noting when they occurred, who performed them, and which patient was involved. These event logs, which reflect the real‐time execution of healthcare processes, provide valuable insights for clinicians, healthcare managers and other decision‐makers in addressing a wide array of process‐related issues within the medical field [11, 14].

Process execution data can be transformed into event logs, which are fundamental for applying process mining algorithms. These event logs capture detailed information about various process instances, referred to as cases, such as the steps taken in treating a specific patient. Each case is composed of a sequence of events, with each event marking the completion of a particular activity within the process. The event log records essential details for each event, including a unique identifier for the case, the activities carried out and the timestamps indicating when these activities occurred. Additionally, event logs may contain supplementary information, such as the type of event, the resource utilised and other attributes that offer a holistic view of the process. These logs are crucial for enabling organisations to analyse and refine their processes by providing deep insights into how these processes are executed in practice [14].

Critical metrics like sojourn time, arrival rate and finish rate play essential roles in identifying the most critical activities within healthcare processes [15, 16]. Sojourn time, also known as waiting time or cycle time, refers to the total time a patient or a case spends within a particular activity or process from start to finish. In healthcare, reducing the sojourn time is critical as it directly impacts patient experience and clinical outcomes. For example, shorter waiting times for diagnostic tests or treatments can lead to faster interventions and improved patient satisfaction. Arrival rate refers to the frequency with which patients or cases enter a particular activity or process within a given time period. High arrival rates can lead to congestion and long waiting times, especially in critical areas like emergency departments. This is crucial for identifying points in the process that are under strain and may require additional resources or re‐engineering to handle the load. Finish rate refers to the rate at which activities or cases are completed within a process. The finish rate directly impacts throughput in healthcare processes. A lower‐than‐expected finish rate in certain activities can indicate inefficiencies, such as inadequate staffing or resource limitations, that need to be addressed [16, 17].

By focusing on these key metrics, healthcare providers can gain a deeper understanding of their processes and identify the most critical activities that require intervention. This approach helps in ensuring that the healthcare system operates efficiently, resources are utilised effectively, and patient outcomes are optimised.

### 2.2 Related Works

In research comparing healthcare organisations, Partington et al. [18] explored patient pathways in four Australian hospitals, with a focus on those entering the emergency department with the suspected acute coronary syndrome. Their analysis covered both the sequence and timing of the processes, evaluating factors such as waiting times, overall duration and length of patient stay. This study revealed key differences, including the more frequent use of angiography—a blood vessel imaging technique—in some hospitals compared to others. Additionally, significant discrepancies in inpatient length of stay were observed across the different hospitals.

Yoo et al. [19] utilised process mining to analyse different time periods to assess the impact of new hospital facilities, such as the consolidation of the cancer centre and clinical neuroscience centre onto the same floor and the introduction of additional administrative counters. By comparing event logs from before and after these changes, the study found that processes in both centres operated more efficiently following the relocation and upgrade of facilities.

Stefanini et al. [20] investigated the emergency department, comparing patient flows and key performance indicators between summer and winter seasons. The study uncovered that during the summer, urgent patients typically faced longer waiting times for their initial consultations compared to the winter period.

Andrews et al. [13] focused on the pre‐hospital care processes for victims of road traffic accidents, dividing the patients into three categories: those who did not require ambulance transport, those transported to local medical centres or elderly care facilities and those who were taken directly to hospitals.

Despite the increasing application of process mining techniques in healthcare, existing research has predominantly focused on analysing broad process flows or patient pathways within various healthcare contexts. However, there is a significant gap in the comparative process mining of sepsis treatment pathways, specifically regarding the identification of critical activities that significantly impact patient outcomes. While previous studies have highlighted differences in patient care processes across institutions and time periods, they often overlook the nuanced, activity‐level variations within sepsis treatment that could inform targeted interventions. Addressing this gap is crucial for optimising sepsis management and improving patient survival rates.

## 3 Methodology

**FIGURE 1:**
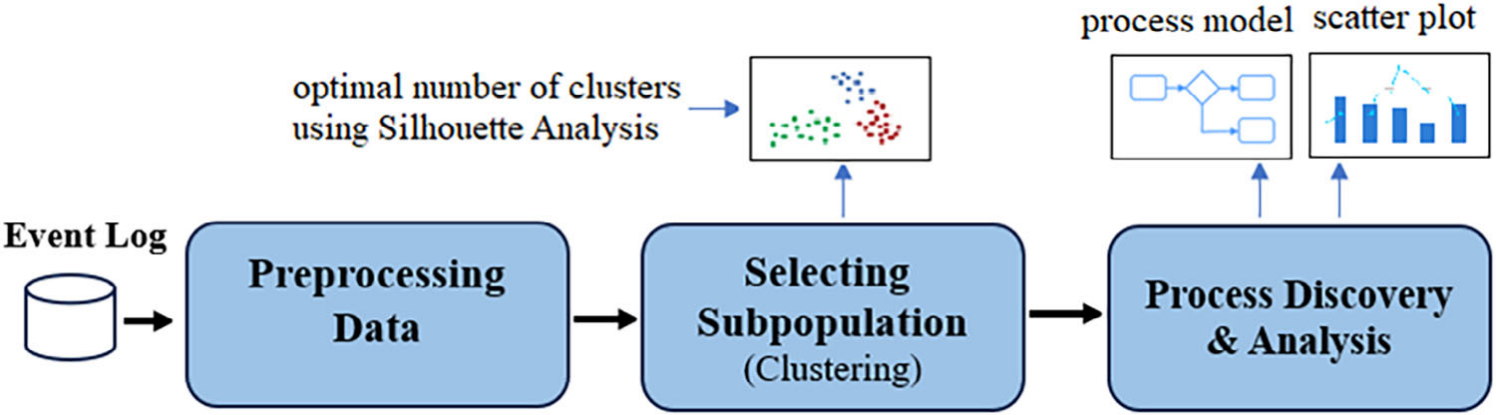
The proposed method.

In this section, we will outline the steps involved in the proposed method, as depicted in Figure 1. The approach utilises tools such as Python and pm4py, a Python library designed for implementing various process mining algorithms.

The dataset utilised in this study was sourced from 4TU.ResearchData (Mannhardt, [21]) and comprises real‐life event logs pertaining to sepsis cases from a hospital setting. Sepsis is a critical, life‐threatening condition usually caused by an infection. Each case in the dataset represents a patient's progression through the hospital system, with events meticulously recorded by the hospital's Enterprise Resource Planning (ERP) system. The dataset contains approximately 1000 cases and a total of 15,000 recorded events spanning 16 distinct activities. Additionally, 39 data attributes were collected, including the group responsible for each activity, test results and information from checklists. To ensure patient confidentiality, all events and their associated attribute values have been anonymised. Although the timestamps of the events have been randomised to protect privacy, the intervals between events within each trace have been preserved to maintain the integrity of the data's temporal sequence.

Referring to Figure 1, the event log associated with the business process is first cleaned and filtered in the initial step. Following this, the data are converted into a data frame to prepare it for the subsequent step, which involves clustering the data for selecting subpopulations. The K‐means algorithm is utilised to cluster the dataset based on four critical features: ‘Age’, ‘SIRSCriteria2OrMore’, ‘DisfuncOrg’ and ‘DiagnosticLacticAcid’. K‐means was chosen for its computational efficiency and its ability to partition data into distinct groups with minimal intra‐cluster variance and maximal inter‐cluster separation [22]. To determine the optimal number of clusters, Silhouette Analysis is applied, which evaluates the quality of clustering by assessing how similar each data point is to its own cluster compared to other clusters, thus providing a measure of cluster cohesion and separation [23].

After identifying the optimal number of clusters through Silhouette Analysis, K‐means clustering was performed to segment the dataset. Each cluster's data were then isolated into separate DataFrames for detailed performance analysis. We employed the PM4PY library for process mining, which offers advanced tools for analysing process performance metrics. The PM4PY library was selected for its robust capabilities in process mining, facilitating a detailed evaluation of process metrics across different clusters [24]. This approach provides critical insights into process performance, enabling targeted improvements based on the characteristics of each patient segment.

## 4 Findings and Discussion

**FIGURE 2:**
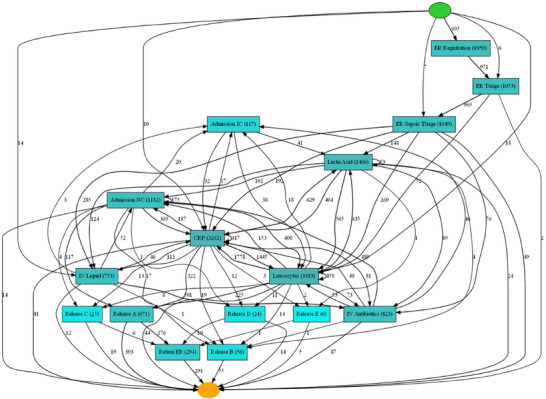
Sepsis process model (Heuristic net).

In this study, the method is applied to a sepsis dataset to showcase its effectiveness, using an event log that captures 16 distinct activities. The process model is depicted in Figure 2 as a Heuristics Net, with the Heuristics Miner algorithm leveraging the Directly‐Follows Graph to manage noise and reveal common patterns (PM4PY 2024). The process, represented as a Petri net, consists of 16 steps categorised into registration and triaging in the emergency ward; measurement of leukocytes, CRP and lactic acid; admission or transfer to care; various discharge procedures and follow‐up visits. ‘ER Registration’ and ‘ER Triage’ are the initial steps in the Sepsis process. Activities related to patient discharge (labelled as Release types A through E) typically occur at the end of the treatment process. The activity ‘Return ER’ follows any form of patient discharge and indicates a subsequent return to the ER. In this model, different colours indicate performance metrics, such as pale blue for activity execution times and bright blue for longer durations. Not all activities occur with the same frequency, with ‘CPR’ and ‘Leucocytes’ being the most frequent, while ‘Release D’ and ‘Release E’ are the least frequent.

Table 1 shows the subpopulation which is divided into three clusters. The second and third columns show the age range and the number of patients for each cluster and the last columns indicate the number of patients who meet the criteria including ‘SIRSCriteria≥2’, ‘DisfuncOrg’, and ‘DiagnosticLacticAcid’.

**TABLE 1:** Clusters of patients.

Cluster number	Age range	Number of case IDs	Number of case IDs meeting the criteria
Cluster 0	75–90	543	34
Cluster 1	20–45	120	1
Cluster 2	50–70	332	20

In this study, we examined critical activities within healthcare clusters by analysing sojourn time, arrival rate and finish rate across three patient groups—Cluster 0, Cluster 1 and Cluster 2. Our analysis identified ‘Return ER’, ‘Admission IC’ and ‘Release C’ as pivotal activities in all clusters, significantly influencing patient flow and outcomes, especially in severe cases like sepsis.

**FIGURE 3:**
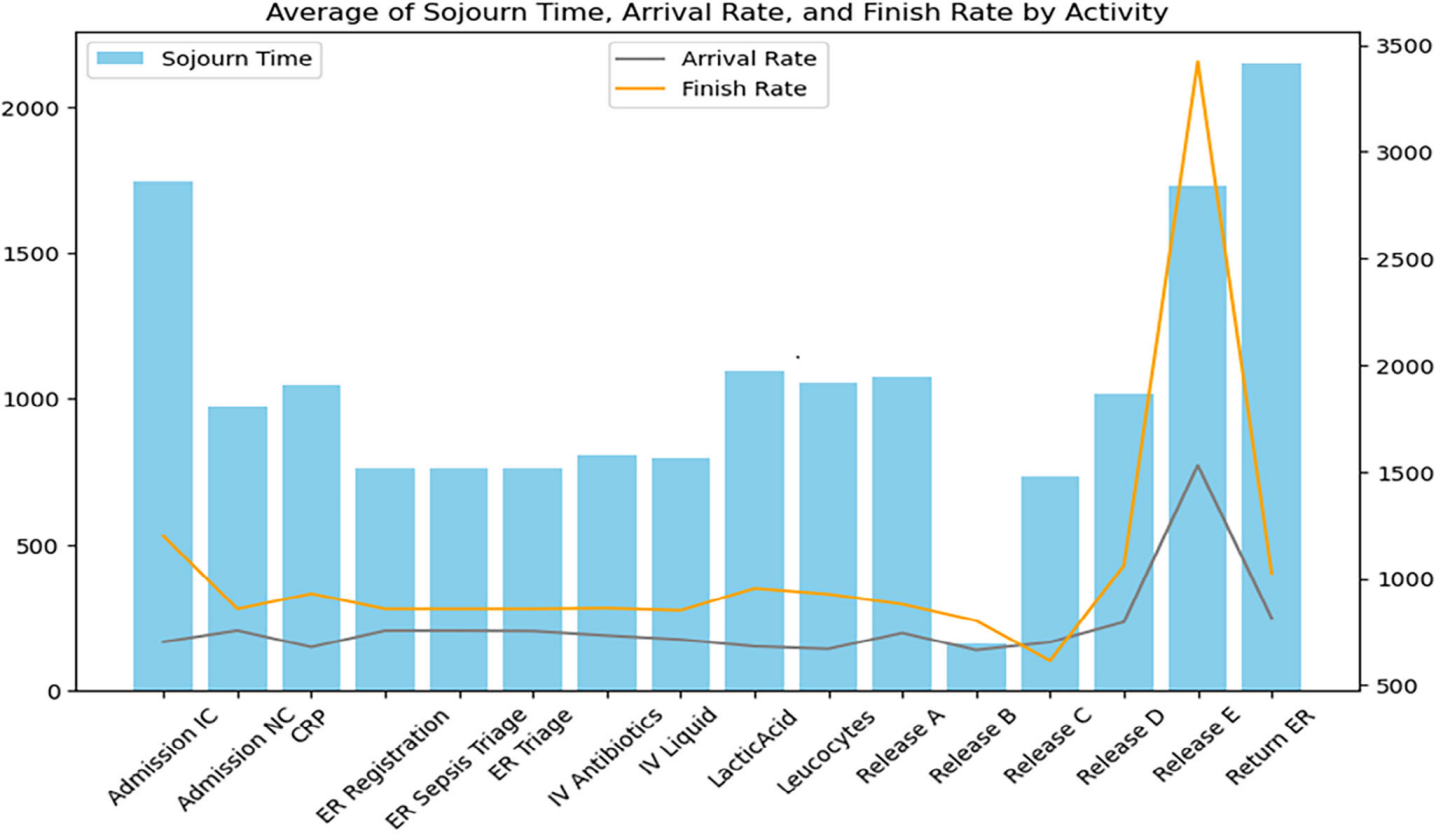
Performance metric—cluster 0.

**FIGURE 4:**
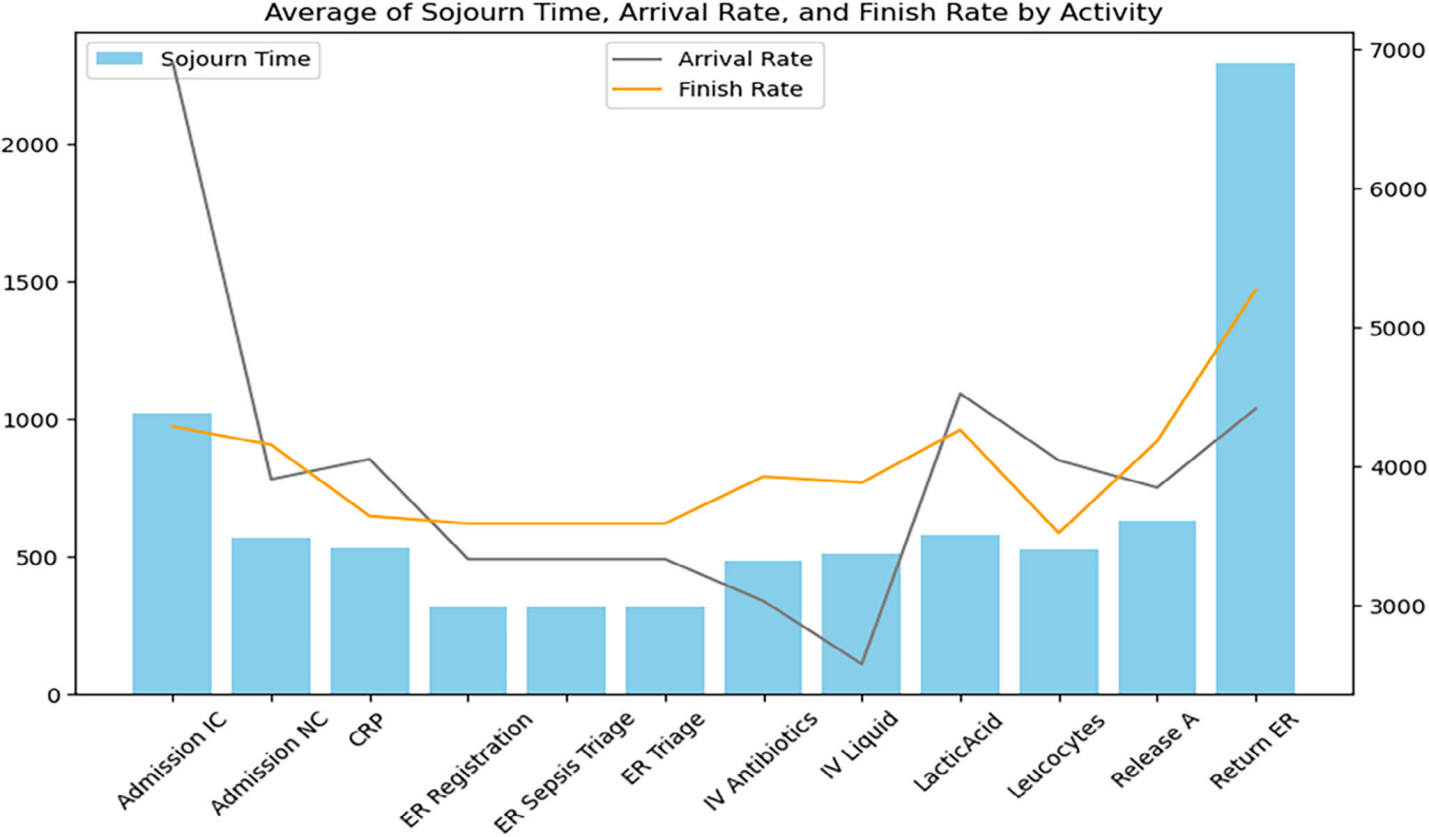
Performance metric—cluster 1.

**FIGURE 5:**
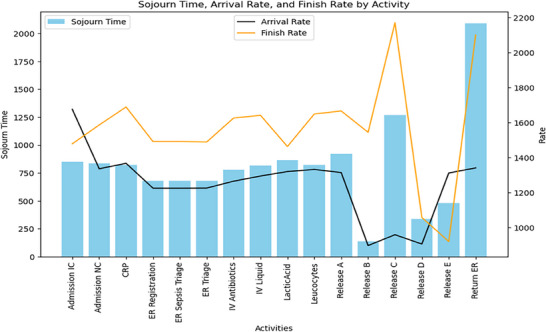
Performance metric—cluster 2.

In Figures 3, 4, 5, the blue bar shows the sojourn time; and the orange and gray lines in the chart show the finish rate and the arrival rate, respectively. Moreover, Tables 2, 3, 4 present key metrics for each cluster highlighting the average sojourn time, arrival rate and finish rate for various activities in the sepsis management process.

**TABLE 2:** Key performance metrics for Cluster 0.

Activity	avg (sojourn time)	avg (arrival time)	avg (finish time)
Admission IC	1746	704	1199
Admission NC	972	757	859
CRP	1046	680	930
ER registration	763	756	859
ER sepsis triage	763	756	859
ER triage	763	755	859
IV antibiotics	807	734	862
IV liquid	796	715	852
Lactic acid	1094	683	954
Leucocytes	1054	671	927
Release A	1078	746	881
Release B	164	666	804
Release C	736	703	617
Release D	1019	797	1061
Release E	1733	1531	3424
Return ER	2152	815	1025

Regarding Table 2 and Figure 3 for cluster 0, Return ER exhibits the highest sojourn time at 2152 units, indicating significant delays, while Release E stands out with a very high finish rate of 3424, despite its lengthy sojourn time of 1733. Activities such as Admission IC and Lactic Acid also show extended sojourn times, suggesting potential bottlenecks or complexities in the care process for this age group.

According to Table 3 and Figure 4 related to cluster 1, ‘Return ER’ shows the highest sojourn time at 2292 units, alongside a notably high finish rate of 5269, indicating significant delays and intensive care needs. ‘Admission IC’ also has an extended sojourn time of 1020, with a high arrival rate of 6909, suggesting potential inefficiencies in the initial admission process for this patient group.

**TABLE 3:** Key performance metrics—cluster 1.

Activity	avg (sojourn time)	avg (arrival time)	avg (finish time)
Admission IC	1020	6909	4289
Admission NC	567	3910	4157
CRP	534	4054	3647
ER registration	316	3337	3592
ER sepsis triage	316	3337	3592
ER triage	316	3337	3592
IV antibiotics	486	3036	3929
IV liquid	509	2583	3886
Lactic acid	577	4525	4265
Leucocytes	524	4046	3524
Release A	631	3850	4183
Return ER	2292	4416	5269

Regarding Table 4 and Figure 5 in cluster 2, ‘Return ER’ has the highest sojourn time at 2092 units, indicating significant delays in this phase. ‘Release C’ shows an extended sojourn time of 1267 and the highest finish rate at 2171, suggesting a complex or time‐consuming process. Other activities, like ‘Admission IC’ and ‘CRP’, have moderate sojourn times with relatively balanced arrival and finish rates, reflecting a steady but potentially improvable process flow for this patient group.

**TABLE 4:** Key performance metrics—cluster 2.

Activity	avg (sojourn time)	avg (arrival time)	avg (finish time)
Admission IC	851	1676	1479
Admission NC	836	1336	1586
CRP	821	1367	1689
ER Registration	681	1225	1491
ER Sepsis Triage	681	1225	1491
ER Triage	679	1226	1489
IV Antibiotics	782	1265	1626
IV Liquid	817	1294	1642
LacticAcid	863	1320	1463
Leucocytes	824	1332	1649
Release A	923	1314	1666
Release B	138	897	1544
Release C	1267	959	2171
Release D	338	905	1057
Release E	478	1312	920
Return ER	2092	1341	2101

### 4.1 Key Findings

#### 4.1.1 ‘Return ER’ as a Consistent Critical Activity

In cluster 0, ‘Return ER’ demonstrated the highest sojourn time (2152) and moderate arrival (814) and finish rates (1025). The prolonged sojourn time indicates that elderly patients, particularly those with severe conditions like SIRS and organ dysfunction, require extensive re‐evaluation or treatment.

In cluster 1, similarly, for younger patients in Cluster 1, ‘Return ER’ had significant sojourn and finish rates, highlighting its critical role in managing acute or deteriorating conditions.

In cluster 2, In the middle‐aged group (Cluster 2), ‘Return ER’ also exhibited the highest sojourn time (2091) with substantial finish rates (2100). This suggests that this activity is crucial for re‐evaluating and stabilising patients before proceeding with their treatment or discharge.

Across all clusters, ‘Return ER’ stands out as a vital activity due to its consistently high sojourn time. This indicates a potential bottleneck where patients spend a significant amount of time, likely due to the severity of their conditions. The moderate to high finish rates suggest that while the process is time‐consuming, it is managed efficiently once initiated. However, the long duration of this activity could lead to delays in care for incoming patients, suggesting the need for resource allocation at this critical stage.

#### 4.1.2 ‘Admission IC’ as a Bottleneck Activity

For cluster 0, ‘Admission IC’ shows a high sojourn time (1746) and significant arrival (704) and finish rates (1198). This suggests that upon admission, elderly patients undergo intensive care and stabilisation, requiring substantial time and resources.

For cluster 1, ‘Admission IC’ had the highest arrival rate (6909) with a notable sojourn time, indicating its importance as a primary entry point for critical care.

For cluster 2, the highest arrival rate (1675) in cluster 2 also corresponds to ‘Admission IC’, reinforcing its role as a key stage in the patient care pathway.

‘Admission IC’ emerges as a bottleneck across all clusters, particularly for elderly and middle‐aged patients. The high arrival rates reflect the activity's importance as the initial point for critical care. However, the substantial sojourn times suggest that delays could occur if the capacity of this activity is not optimised. Ensuring that ‘Admission IC’ is adequately staffed and resourced could reduce these delays, leading to improved patient throughput and outcomes.

#### 4.1.3 ‘Release C’ and Complexity in Discharge Processes

For cluster 0, ‘Release E’ (closely related to ‘Release C’ in other clusters) showed a significant sojourn time (1733) and an extremely high finish rate (3424), indicating a critical final evaluation before patient discharge.

For cluster 1, ‘Release C’ displayed considerable sojourn times with a notable finish rate, highlighting the thoroughness required in discharging patients, particularly in ensuring all health criteria are met.

For cluster 2, ‘Release C’ also had high sojourn (1267) and finish rates of about 2170, suggesting complexity in the discharge process, possibly involving detailed final assessments or additional treatment before discharge.

The discharge process, represented by ‘Release C’, is complex and requires careful management to ensure that patients are ready to leave the healthcare facility safely. The high sojourn and finish rates suggest that while this process is thorough, it might also be a point where delays could occur, particularly if all necessary evaluations are not conducted efficiently. Improving the efficiency of the discharge process, without compromising care quality, could enhance patient satisfaction and reduce the risk of readmission.

The high sojourn times in activities like ‘Return ER’ and ‘Admission IC’ across all clusters suggest that these stages are potential bottlenecks in patient flow. Addressing these bottlenecks could significantly reduce delays in care, thereby improving overall process efficiency and patient outcomes.

The findings indicate that activities with high sojourn times and arrival rates, such as ‘Admission IC’ and ‘Return ER’, require more resources. This could include additional staffing, equipment or expanded facilities to handle the patient load more efficiently.

Prolonged sojourn times in critical activities can lead to delayed treatment, increased patient stress and higher risks of complications. By optimising these critical stages, healthcare providers can ensure timely interventions, which are crucial for improving patient outcomes, particularly in vulnerable populations such as the elderly.

Therefore, the analysis of sojourn time, arrival rate, and finish rate across different patient clusters reveals that ‘Return ER’, ‘Admission IC’ and ‘Release C’ are the most critical activities in healthcare processes. These activities consistently show high sojourn times and arrival rates, indicating their importance in managing patient care, particularly for those with severe conditions.

To improve healthcare outcomes, it is essential to focus on these critical activities by optimising resource allocation, reducing bottlenecks, and ensuring efficient patient processing through these stages. This approach will not only enhance the quality of care but also improve the overall efficiency of healthcare delivery, particularly for patients with complex or severe conditions.

## 5 Conclusion

This study presents a detailed examination of sepsis care pathways through the application of advanced comparative process mining techniques. By focusing on critical performance indicators such as sojourn time, arrival rate and finish rate, we have identified significant bottlenecks at key process points—specifically ‘Return ER’, ‘Admission IC’, and ‘Release C’. These activities consistently emerged as areas of delay across various patient clusters, underscoring their pivotal role in influencing the efficiency and effectiveness of sepsis management. The identification of these bottlenecks is particularly critical in the context of sepsis, where timely intervention is paramount to patient survival and recovery. By highlighting these process inefficiencies, our findings offer a targeted framework for healthcare providers to refine patient flow, optimise resource allocation and ultimately reduce sepsis‐related mortality and readmission rates. This work not only contributes to the understanding of sepsis trajectories but also provides actionable insights for the continuous improvement of sepsis care protocols in hospital settings.

Despite the insights gained, this study has several limitations. First, the dataset is limited to a single hospital, which may not fully represent sepsis trajectories in other healthcare settings. Additionally, the event logs used are anonymised and randomised, potentially impacting the accuracy of temporal patterns and the generalisability of the findings. The clustering method used, K‐means, while effective, may not fully capture the complex, nonlinear relationships between variables that could influence patient outcomes. Furthermore, the study focuses on specific metrics (sojourn time, arrival rate and finish rate) and may overlook other critical factors, such as patient comorbidities, that could influence the sepsis treatment process.

Future research should address these limitations by expanding the analysis to include multiple hospitals and a more diverse patient population. Incorporating other clustering techniques, such as hierarchical clustering or deep learning‐based approaches, could provide deeper insights into patient subgroups. Moreover, integrating additional metrics, like resource utilisation or patient comorbidities, could offer a more holistic view of the factors affecting sepsis outcomes. Longitudinal studies could also be conducted to assess the long‐term impact of interventions targeted at the identified bottlenecks. Finally, real‐time process mining could be explored to enable dynamic adjustments to sepsis management protocols, potentially improving patient outcomes even further.

## Author Contributions

**Mohsen Mohammadi**: conceptualization, data curation, formal analysis, funding acquisition, investigation, methodology, project administration, resources, software, supervision, validation, visualization, writing – original draft, Writing – review & editing.

## Conflicts of Interest

The author declares no conflicts of interest.

## Data Availability

The data that support the findings of this study are openly available at https://data.4tu.nl/articles/dataset/Sepsis_Cases_‐_Event_Log/12707639.
